# ETV5 regulates proliferation and cell cycle genes in the INS‐1 (832/13) cell line independently of the concentration of secreted insulin

**DOI:** 10.1002/2211-5463.13724

**Published:** 2023-11-02

**Authors:** Yael E. Díaz‐López, Gloria Erandi Pérez‐Figueroa, Vicenta Cázares‐Domínguez, María E. Frigolet, Ruth Gutiérrez‐Aguilar

**Affiliations:** ^1^ División de Investigación, Facultad de Medicina Universidad Nacional Autónoma de México (UNAM) México; ^2^ Laboratorio de Investigación en Enfermedades Metabólicas: Obesidad y Diabetes Hospital Infantil de México “Federico Gómez” México; ^3^ Unidad de Investigación en Inmunología y Proteómica Hospital Infantil de México “Federico Gómez” México

**Keywords:** *E2F1*, E‐twenty‐six variant 5, INS‐1 (832/13), insulin secretion, *p27* ^*Cdkn1b*^, proliferation

## Abstract

The transcription factor E‐twenty‐six variant 5 (ETV5) regulates acute insulin secretion. Adequate insulin secretion is dependent on pancreatic β‐cell size and cell proliferation, but the effects of ETV5 on proliferation, cell number, and viability, as well as its relationship with insulin secretion, have not been established yet. Here, we partially silenced *ETV5* in the INS‐1 (832/13) cell line by siRNA transfection and then measured secreted insulin concentration at different time points, observing similar levels to control cells. After 72 h of *ETV5* silencing, we observed decreased cell number and proliferation, without any change in viability or apoptosis. Thus, partial silencing of *ETV5* modulates cell proliferation in INS‐1 (832/13) independently of secreted insulin levels via upregulation of *E2F1* and of inhibitors of the cyclin/CDKs complexes (*p21* ^*Cdkn1a*^, *p27* ^*Cdkn1b*^, and *p57* ^*Cdkn1c*^) and downregulation of cell cycle activators (*PAK3* and *FOS*).

## Graphical abstract

The transcription factor ETV5 promotes cell proliferation in the INS‐1 (832/13) cell line by regulating gene expression of *E2F1*, cyclin/CDK inhibitors (*p21* ^*Cdkn1a*^, *p27* ^*Cdkn1b*^, and *p57* ^*Cdkn1c*^), and cell cycle activators (*PAK3* and *FOS*). This effect is independent of the concentration of secreted insulin.

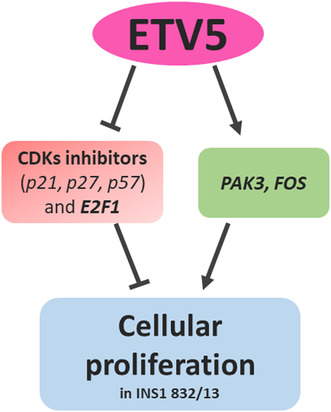

**Abbreviations**
CCND1: cyclin D1
CCND2: cyclin D2
CDK: cyclin‐dependent kinase
E2F1: E2F transcription factor 1
ETV5: E‐twenty‐six variant 5
PAK3: p21 activated kinase 3
RB: retinoblastoma protein

The transcription factor E‐twenty‐six variant 5 (ETV5) belongs to the E‐twenty‐six family (ETS) that is integrated with 28 transcription factors and sub‐classified in the polyomavirus enhancer activator 3 group (PEA3), due to its structural composition [1, 2].

The ETV5 knockout mice have reduced body weight, body fat mass, and impaired glucose tolerance, due to decreased insulin secretion. Moreover, ETV5 KO mice have a smaller pancreas, reduced islet area, and decreased pancreatic β‐cell size [3]. In addition, *in vitro* studies using the INS‐1 (832/13) cell line and human pancreatic β cells, we demonstrated that ETV5 silencing provoked a reduced insulin secretion by transcriptional regulation of exocytotic genes by ETV5 [3]. Recently, *ETV5* was identified as a target of microRNA‐200c in type 2 diabetic human islets, provoking reduction in *ETV5* expression and leading to reduced secretion [4].

An adequate insulin secretion depends on an appropriate pancreatic β‐cell mass, given by a correct β‐cell size and number of cells [5, 6]. In turn, it is well known that insulin is able to activate signaling pathways involved in the proliferation of pancreatic β cells and, thus, contributes to the β‐cell mass [7]. Previously, a tendency toward reduction in β‐cell mass and a decrease in cell size was described in the ETV5 KO model [3]. However, to date no studies have studied the influence of ETV5 on cell proliferation in a β‐cell line.

The ETV5 has been reported to be overexpressed in different types of human cancer and its involvement in cell proliferation has been proven [8, 9, 10]. Cell proliferation is mediated by genes implicated in the cell cycle. During the G1 phase of the cell cycle, cyclin D binds to cyclin‐dependent kinase 4 (CDK4), generating a complex that phosphorylates the retinoblastoma protein (Rb)‐E2F complex [11]. While Rb is bound to E2F, this complex inhibits E2F activity as a transcriptional factor. However, Rb phosphorylation, given by the CDK‐cyclin D complexes, releases E2F, which allows the transcription of genes required for DNA synthesis, enabling the transition from the G1 to the S phase [12].

In addition, CDK‐cyclin complexes are modulated by CDK inhibitors, allowing a strict regulation of the cell cycle advance. Cell cycle inhibitors as *p21* ^*Cdkn1a*^, *p27* ^*Cdkn1b*^, and *p57* ^*Cdkn1c*^ are able to bind to the CDK‐cyclin complexes, inhibiting its kinase action [13]. On the contrary, E2F1 is capable of transcriptional upregulate the expression of *p27* ^*Cdkn1b*^ and *p21* ^*Cdkn1a*^ by direct binding to its promoter region, causing cell cycle arrest [14, 15]. Moreover, *PAK3* has been reported to regulate cell cycle and differentiation in β cells of embryonic pancreas [16]. Additionally, the protooncogene *FOS* can control cell cycle reentry or progression [17].

It is known that ETV5 regulates the transcription of *cyclin D1* and *cyclin D2* in thyroid cancer cells [18]. Moreover, it has previously been published that *ETV5* contributes to tumor growth and progression in colorectal cancer, by binding directly to *p21* ^*Cdkn1a*^ promoter repressing its expression and upon *ETV5* knockdown cell growth slows down, due to the inhibition of the repression of p21^Cdkn1a^ [19]. In embryonic stem cells, the *Etv4* and *Etv5* double knockout was associated with decreased cell number and proliferation, in contrast to control cells. In this model, CDK inhibitors (*p19*, *p15*, and *p57*) were overexpressed, leading to reduced cell proliferation [20]. Recently, potential ETV5 targets were investigated in human islets using siC or siE and performing an RNA‐Seq analysis, finding *p21* ^*Cdkn1a*^ and *p57* ^*Cdkn1c*^ overexpressed, and downregulation of *PAK3 and FOS* [4]. However, the role of ETV5 on proliferation and its target genes *E2F1*, *p21* ^*Cdkn1a*^, *p27* ^*Cdkn1b*^, p*57* ^*Cdkn1c*^, *PAK3*, and *FOS* has never been explored in the INS‐1 (832/13) cell line.

Thus, the aim of this study was to analyze the effect of ETV5 on cell proliferation in the INS‐1 (832/13) cells. Our results showed that partial silencing of *ETV5* reduces cell proliferation independent of secreted insulin levels in the cell line model INS‐1 (832/13). In addition, *ETV5* silencing upregulated *E2F1 and cell cycle inhibitors* (*p21* ^*Cdkn1a*^, *p27* ^*Cdkn1b*^, and *p57* ^*Cdkn1c*^) and downregulated cell cycle activators (*PAK3* and *FOS*), elucidating the mechanism through which cell cycle is arrested. Therefore, ETV5 plays an important role in metabolism regulating cell proliferation in pancreatic β cells.

## Materials and methods

### Cell culture

INS‐1 (832/13) cell line was donated by Dr Newgard of Duke University Medical Center and used between passages 20 and 34. Cells were thawed (2 weeks before starting transfection assays) and splitted every third day. Cell cultures were incubated in RPMI‐1640 (R8758, SIGMA, Burlington, MA, USA) with 10% heat‐inactivated fetal bovine serum (HI‐FBS, 10082, GIBCO, Grand Island, NY, USA), 10 mm HEPES (15630‐080, GIBCO), 2 mm l‐Glutamine (25030‐081, GIBCO), 1 mm sodium pyruvate (S8636, SIGMA), 50 μm 2‐mercaptoethanol (21985023, GIBCO), and penicillin (100 U·mL^−1^)‐streptomycin (10 mg·mL^−1^) (SV30010, HyClone, Logan, UT, USA). Incubation conditions were damped ambient at 37 °C and 5% of CO_2_.

### Transfection

INS‐1 (832/13) cells were plated in a 6‐well plate at 6 × 10^5^ cells per well. After 24 h, these cells were transfected with 20 nm ETV5 siRNA (siE, using a pool of 4 different siRNAs to silence ETV5, SMARTpool, L‐087219‐02, Dharmacon; Lafayette, CO, USA) or 20 nm control siRNA (siC, D‐001810‐10‐05, Dharmacon), using 1 μL of transfection reagent (T‐2001‐02, Dharmaphect; Thermo Scientific, Waltham, MA, USA) for each transfection, according to the manufacturer's instructions.

### Gene expression

Total RNA was extracted from INS‐1 (832/13) cells using Jena Bioscience RNA extraction kit (PP‐210S, Jena, Erfurt, Germany), and the cDNA was synthesized using Jena Bioscience SCRIPT cDNA synthesis kit (PCR‐511S; Jena). qPCR was performed using the AriaMX PCR system (Malaysia, Singapore) through TaqMan assays (Thermo Scientific). Relative mRNA expression for *ETV5* (Rn00465814_g1), *CCND2* (cyclin D2) (Rn03020897_m1), *S6K1* (Rn00579546_m1), *E2F1* (Rn01536222_m1), *p27* ^*Cdkn1b*^ (Rn00582195_m1), *p21* ^*Cdkn1a*^ (Rn00589996_m1), *p57* ^*Cdkn1c*^ (Rn01502044_g1), *PAK3* (p21‐activated kinase 3, Rn00693022_m1), and *FOS* (Rn02396759_m1) was calculated relative to *L32* (Rn00820748_g1) housekeeping gene, using ΔΔ*C*_t_ method. Values presented are relative expression to the cells transfected with control siRNA (siC).

### Cell count and representative images (daily)

**Fig. 1:**
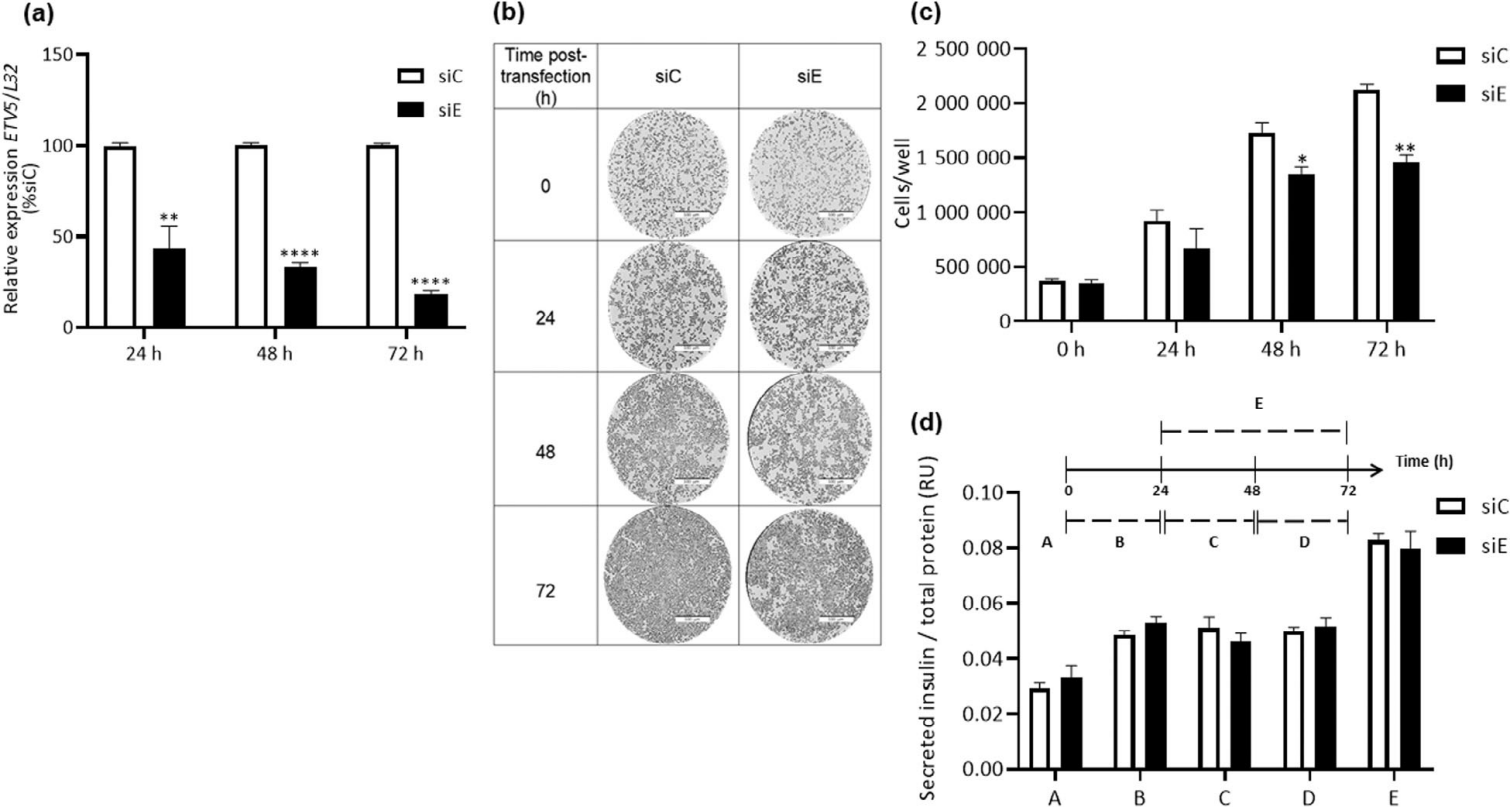
Partial E‐twenty‐six variant 5 (*ETV5*) silencing provokes reduced cell number independently of secreted insulin concentration in INS‐1 (832/13) cell line. (a) mRNA *ETV5* relative expression after control siRNA (siC) and *ETV5* siRNA (siE) transfection assays at 24, 48, and 72 h post‐transfection. (b) Representative images (10×) of growing culture cells siC and siE transfection at 0, 24, 48, and 72 h post‐transfection. Scale bars: 100 μm. (c) Cell number in siC and siE treatments at 0, 24, 48, and 72 h post‐transfection. (d) Secreted insulin concentration normalized to total protein at different time points after siC or siE transfection: at 0 h (A), 24 h (B), from 24 to 48 h (C), from 48 to 72 h (D), and from 24 to 72 h (E). Two‐way ANOVA, *P < 0.05, **P < 0.001, ****P < 0.00001, *n* = 4. All results are presented as the mean ± SD.

INS‐1 (832/13) cells were plated in a 12‐well plate at 3 × 10^5^ cells per well. The next day, the cells were transfected with 20 nm siC or siE and counted (time 0). Then, after 24, 48, and 72 h, the cells were counted using a Neubauer chamber. In addition, the cells were also photographed (10×, 100 μm) at the same time points, and only representative images are presented in Fig. 1.

### Determination of secreted insulin concentration

An aliquot of secreted insulin to the cell media was recovered at different time points after siC or siE transfection: 0 h (before the transfection) and every 24 h, before adding fresh media to the INS‐1 cell culture every time point (Fig. 1d, time points B, C, and D). For Fig. 1d time point E, the media was changed 24 h post‐transfection and then left for the rest of the experiment, having a total of 48 h of insulin concentration accumulation. Secreted insulin concentration was measured by immunodetection with an ELISA kit (80‐INSRT‐E01; ALPCO, Salem, NH, USA) according to the manufacturer's instructions. Total protein concentration was measured using DC Protein Assay (Bio‐Rad, Hercules, CA, USA). We report secreted insulin concentration normalized by total protein concentration (relative units, RU).

### Determination of proliferation (daily)

INS‐1 (832/13) cells were plated and transfected with siC or siE on coverslips. After 24, 48, and 72 h of transfection, cells were incubated for 3 h with 5 μm of 5‐ethynyl‐2′‐deoxyuridine (EdU). Then, they were fixed for 15 min with 3.7% formaldehyde solution in PBS and permeabilized for 20 min with 0.5% Triton X‐100 solution in PBS. The EdU incorporated into cells was bound to 5/6‐sulforhodamine 101‐PEG3‐azide fluorochrome, as recommended by the manufacturer (EdU‐Click 594, Sigma‐Aldrich, Burlington, MA, USA). DAPI dye (ab104139, Abcam, Cambridge, UK) was used to visualize the nuclei. Then, samples were photographed by fluorescence microscopy (20×, 50 μm) and analyzed. To report EdU‐positive cells, these were normalized to total cells labeled with DAPI.

### MTT proliferation assay (daily)

INS‐1 (832/13) cells were plated in a 12‐well plate at 3 × 10^5^ cells per well. Cell proliferation was measured by treating the cells with yellow MTT (3‐(4,5‐dimethilthiazol‐2‐yl)‐2,5‐diphenyltetrazolium bromide, a tetrazole), which is reduced to purple formazan in the mitochondria of proliferating cells. The absorbance of the colored solution was measured at 590 and 620 nm by a spectrophotometer. The MTT proliferation assay was performed and measured at 24, 48, and 72 h, according to the manufacturer's instructions (product No. M2128; Sigma‐Aldrich, Saint Louis, MO, USA).

### Determination of viability

Seventy‐two hours post‐transfection, cells were trypsinized and an 80 μL aliquot containing 5 × 10^4^ cell·mL^−1^ of each treatment was diluted in PBS supplemented with 2 μm EDTA and 3% FBS. That suspension was mixed with 150 μL of viability reagent (MCH100102, Luminex, Austin, TX, USA) and incubated for 5 min. The samples were read in the cell analyzer Muse^®^ (Austin, TX, USA). The viability profiles were obtained by taking into consideration the cell size index (adjusted to 1) and viability, and the percentage of live and dead cells was calculated.

### Determination of apoptosis

Seventy‐two hours post‐transfection, cells were trypsinized and a 100 μL aliquot containing 5 × 10^4^ cell·mL^−1^ was mixed with 100 μL of annexin‐V and dead cell reagent (MCH100105, Luminex). The samples were incubated for 20 min at room temperature in the dark and then read in cell analyzer Muse®. Apoptosis profiles were obtained considering the cell size index (adjusted to 1) and annexin‐V index, and the total percentage of apoptotic cells was determined.

### Western blot

Proteins were extracted from the samples using RIPA buffer (89900, Thermo Scientific) with 1% protease inhibitor cocktail (78410, Thermo Scientific), 1% phosphatase inhibitor cocktail (1862495, Thermo Scientific), and 0.1% benzonase (E1014‐25KU, Sigma). Proteins were quantified using Bio‐Rad protein quantification kit (500‐0116, Bio‐Rad). The samples were separated on 12% gradient SDS/PAGE gels using 75 μg per well of protein extract. Gels were transferred to PVDF membranes and were blocked in PBS solution with 5% milk and 1.2% Tween, for 1 h. Specific proteins were identified with primary antibodies ETV5 (dilution 1 : 200, sc‐100941, Santa Cruz Biotechnology, Dallas, TX, USA), CCND2 (cyclin D2) (dilution 1 : 7000, GTX32545, GeneTex, Irvine, CA, USA), E2F1 (dilution 1 : 100, sc‐56,662, Santa Cruz Biotechnology), p27^Cdkn1b^ (dilution 1 : 400, OALA04950, Aviva Systems Biology, San Diego, CA, USA), and β‐actin as loading control (dilution 1 : 5000, 4967S; Cell Signaling, Danvers, MA, USA). Secondary antibodies were anti‐mouse IgG (dilution 1 : 5000, 7076P2; Cell Signaling) and anti‐rabbit IgG (dilution 1 : 5000, 7074P2; Cell Signaling). Finally, protein bands were visualized by enhanced chemiluminescence reagent (WBKLS0100, Millipore, Burlington, MA, USA). The densitometry of the images was processed through ImageJ v1 software 52o, and this was reported as relative units of densitometry.

### Statistical analyses

Results are presented as mean ± SD. Results were analyzed by one‐way ANOVA (*post hoc* Tukey), two‐way ANOVA, or Student's *t*‐tests, where appropriate. The level of significance was set as *P* < 0.05. Data were analyzed with graphpad prism, version 8.0.2, GraphPad by Dotmatics, Boston, MA, USA.

## Results

### Partial silencing of *ETV5* decreases cell number independently of secreted insulin levels in the INS‐1 (832/13) cell line

Our group had previously described the implication of ETV5 on insulin secretion by regulating the transcription of genes involved in insulin exocytosis, using the INS1 (832/13) cell line [3]. ETV5 has been implicated in proliferation in different type of cancer cells [18, 19]. However, we wanted to pursue whether there are other mechanisms in which ETV5 could regulate insulin secretion, such as cell proliferation. Therefore, we transfected INS‐1 (832/13) cells with siRNA control (siC) or siRNA against ETV5 (siE) and analyze the silencing of the gene every 24 h. After 24 h of ETV5 siRNA transfection, *ETV5* transcript was reduced to 43% expression at 48 h to 33%, and at 72 h we obtained the lowest gene expression of 18% (Fig. 1a).

We then analyzed cell confluence and cell number every 24 h. We noticed that *ETV5* silencing provoked a decrease in cell confluence that was noticeable under the microscope (10×) at 48 and 72 h post‐transfection. To verify the reduced cell confluence, cell count was performed showing reduction of 21% at 48 h and 32% at 72 h post‐transfection, compared with control cells (Fig. 1b,c). These results indicate that *ETV5* modifies cell number in INS‐1 (832/13) cells.

In our previous paper, we showed in a glucose‐stimulating insulin secretion experiment that after 72 h post‐transfection of siC or siE, partial silencing of ETV5 provoked a reduced insulin secretion after 1 h of glucose stimulation in INS‐1 (832/13) cells [3]. In Fig. 1, we demonstrated a reduced cell number at 72 h post‐transfection with siE.

It is well known that insulin can act as a mitogen and a variation of its concentration can modify cell number and proliferation [21]. For this reason, secreted insulin concentration was measured to verify whether a variation in secreted insulin concentration could explain the changes in cell number. Interestingly, partial silencing of *ETV5* cells and control cells showed no difference in secreted insulin concentration after 24, 48, or 72 h of glucose stimulation post‐transfection (Fig. 1d). Therefore, this assay proves that the decreased cell number is independent of secreted insulin concentration.

### Partial silencing of *ETV5* modifies cell proliferation without changing viability or apoptosis in INS‐1 (832/13) cell line

Possible explanations for a lower cell number on siE transfected cells are a decrease in cell proliferation, a loss of viability, or an increase in apoptosis. Therefore, all three conditions were evaluated.

**Fig. 2:**
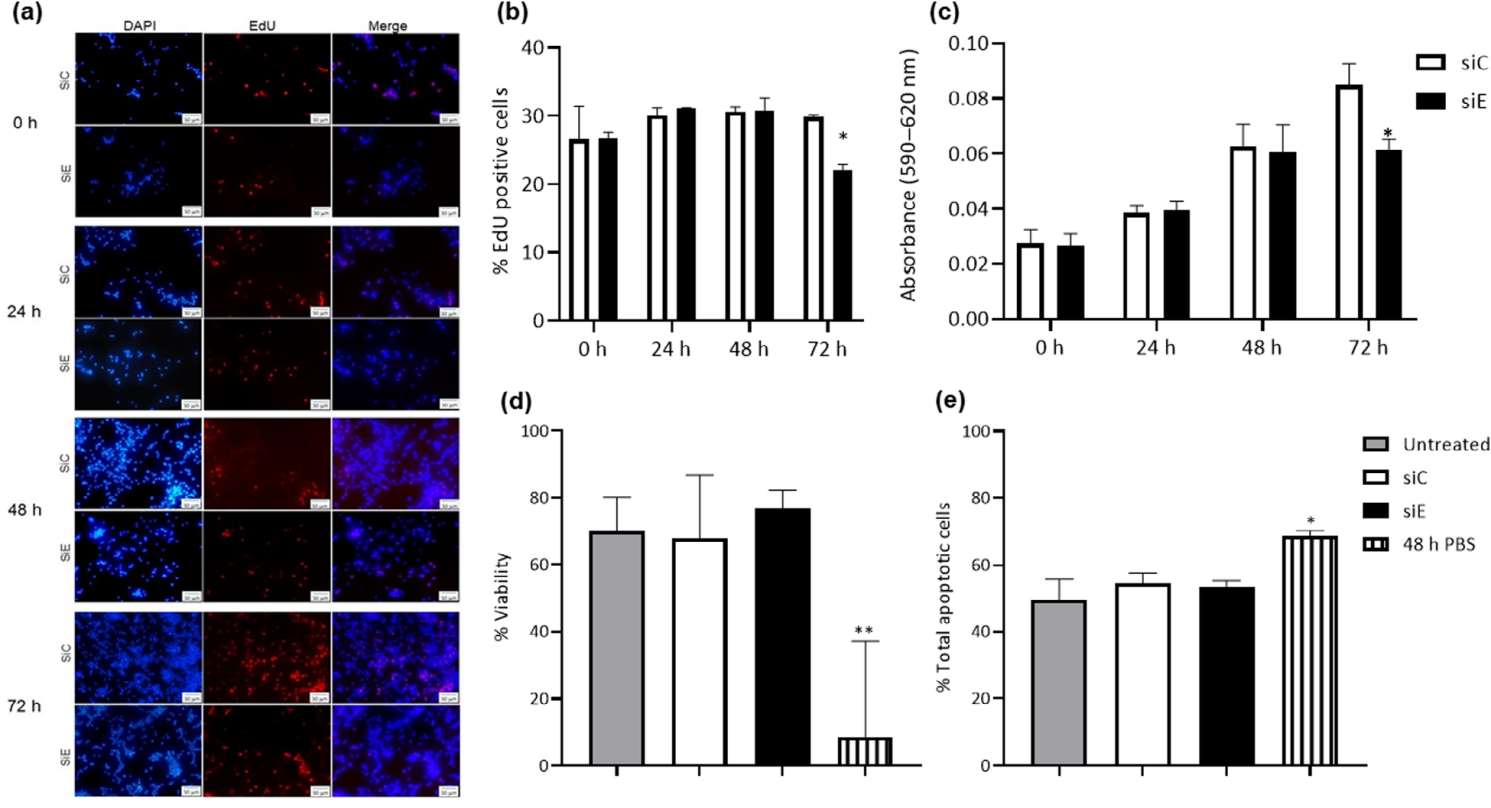
E‐twenty‐six variant 5 (*ETV5*) silencing reduces cell proliferation in INS‐1 (832/13) cell line and does not affect viability and apoptosis. (a) Percentage of positive EdU proliferating cell (% ratio proliferating cells divided by the total cell number) at 0, 24, 48, and 72 h post‐transfection with siC or siE. Two‐way ANOVA, *P < 0.05, *n* = 3. All results are presented as the mean ± SD. (b) Representative images of cell proliferation determination by fluorescence microscopy (20×), DAPI labeled in blue, EdU labeled in red, after 0, 24, 48, and 72 h post‐transfection with siC or siE. Scale bars: 50 μm. (c) MTT (3‐(4,5‐dimethilthiazol‐2‐yl)‐2,5‐diphenyltetrazolium bromide) assay, after 0, 24, 48, and 72 h post‐transfection with siC or siE. One‐way ANOVA *P < 0.05. *n* = 4. (d) Viability percentage after 72 h of post‐transfection with siC or siE. One‐way ANOVA **P < 0.001, *n* = 3, (e) Percentage of apoptotic cells after 72 h of post‐transfection with siC or siE. One‐way ANOVA *P < 0.05, *n* = 3. For (d and e), gray columns represent control cells without transfection (untreated); white columns represent cells transfected with control siRNA (siC); black columns represent cells transfected with ETV5 siRNA (siE), and vertical lines columns represent control cells incubated 48 h in PBS. All results are presented as the mean ± SD.

Interestingly, a time‐course evaluation of cell proliferation was performed after 24, 48, and 72 h post‐transfection. DAPI‐labeled cells showed a visually decreased cell confluence with partial absence of *ETV5* in comparison with control cells at 72 h post‐transfection under the microscope (20×) (Fig. 2a). To further demonstrate that cell proliferation was decreased during partial absence of *ETV5*, the percentage of proliferating cells was calculated using the EdU incorporation assay and the total number of cells visualized by DAPI. EdU is a thymidine analog that is incorporated into DNA during its synthesis and facilitates evidencing of existing proliferating cells. Results show that the siE transfected cells were not different at 24 and 48 h post‐transfection; however, 72 h post‐transfection, there was a 25.8% reduction in proliferating cells with respect to control (Fig. 2b).

Moreover, to further prove that the reduced number of cells in the absence of ETV5 was provoked by a reduced cell proliferation, we performed the MTT proliferation assay, since this technique would confirm our results regarding proliferation and the degree in which the process would be modified. This assay is based on the transformation of tetrazolium salts (MTT) to colored formazan by the nicotinamide‐adenine‐dinucleotide (NAD(P)H) coenzyme and dehydrogenases from metabolically active and proliferating cells [22]. The MTT assay showed a similar result to the EdU assay, where no difference was noticed at 24 and 48 h, but with a significant reduction in proliferation (28.23%) of siE‐treated cells compared with siC 72 h post‐transfection (Fig. 2c). The reduction in cellular proliferation was similar in both assays (25% with EdU and 28.23% with MTT); therefore, proving by two different approaches that ETV5 is involved in cell proliferation.

Then, we also needed to understand whether there were other mechanisms, such as viability and apoptosis, provoking cell reduction in the absence of ETV5 at 72 h post‐transfection of siC or siE. Viability was assessed by adding a dye; viable cells exclude the viability reagent containing the dye, while dead cells internalize the reagent. Thus, the number of viable versus dead cells can be evaluated with this method. We observed that no differences were observed between siE, siC, and untreated cells. However, cells starved for 48 h in PBS showed reduced cell viability (Fig. 2d).

To measure apoptosis, we added a reagent containing Annexin‐V that binds to phosphatidylserine (PS), which is externalized in dying cells. Also, it contains 7‐aminoactinomycin D, an indicator of cell membrane structural integrity, which allows to distinguish early apoptotic cells (not permeable) from dead cells (permeable). The results showed that there is no difference between siE, siC, and untreated cells. However, cells starved for 48 h in PBS showed an increase in total apoptotic cells (Fig. 2e).

Thus, the lower cell number observed in the partial absence of *ETV5* was due to a reduction in cell proliferation.

### Partial silencing of ETV5 modifies the expression of *E2F1* , *p27* ^*Cdkn1b*^ , *p21* ^*Cdkn1a*^ , *P57* ^*Cdkn1c*^ , *PAK3* , and *FOS* in INS‐1 (832/13) cell line

In a previous report, a transcriptome of muscle of *ETV5 KO* mice showed differential expression of several genes implicated in cell cycle [23]. From those genes, we selected some and performed *in silico* analysis, finding that cell cycle genes, such as *CCND2* (*cyclin D2*), *S6K1*, *E2F1*, and *p27* ^*Cdkn1b*^ contain ETV5 binding sites to their promoters (analysis performed by Genomatix, data not shown). Moreover, potential ETV5 targets were investigated in human islets by RNA‐Seq analysis [4]. Among 58 genes that the authors found differentially expressed between control and *ETV5* silenced islets, we chose genes related to cell cycle control (*p21* ^*Cdkn1a*^, *p57* ^*Cdkn1c*^, *PAK3*, and *FOS*) and we performed qPCR in our cell‐line model.

**Fig. 3:**
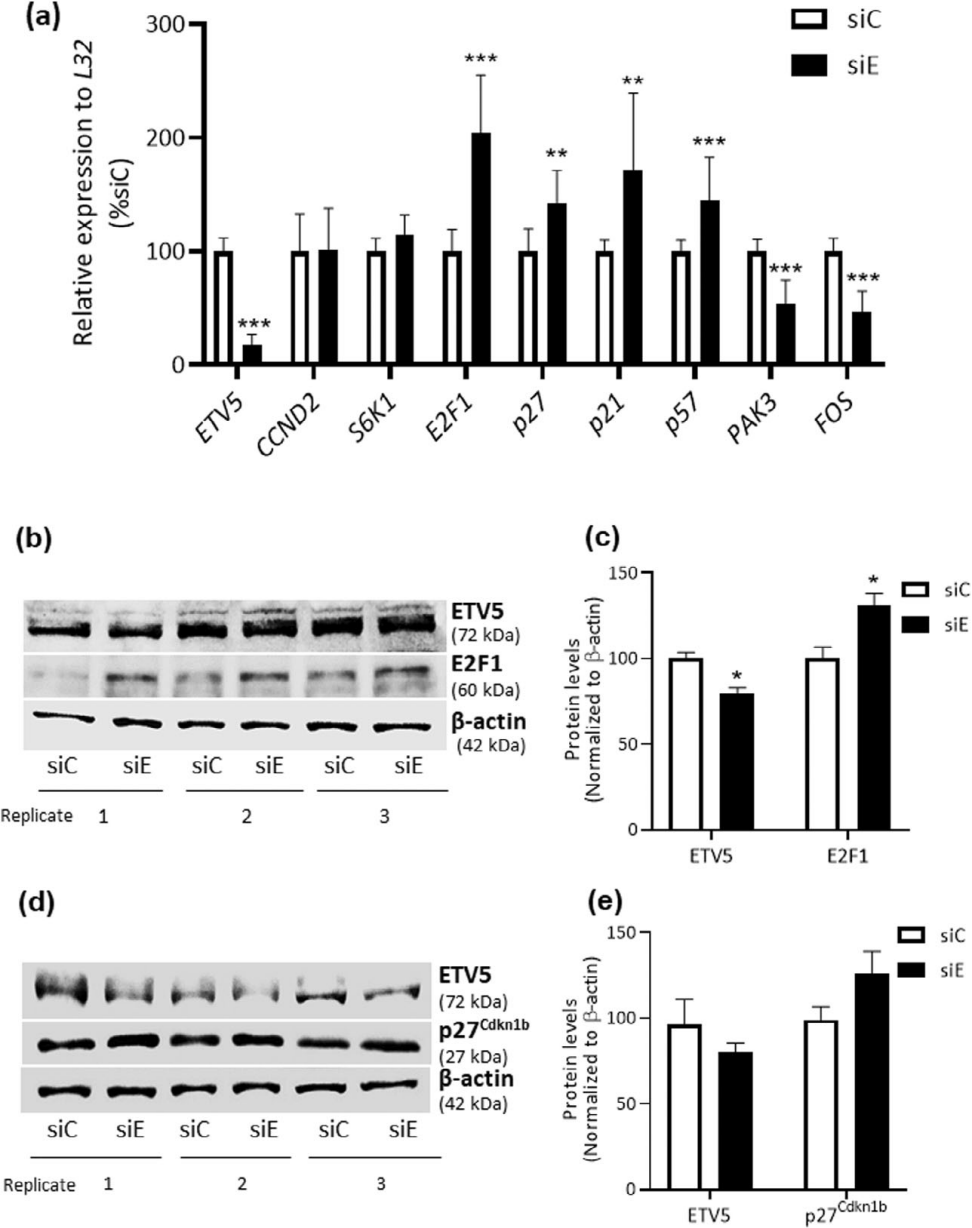
E‐twenty‐six variant 5 (*ETV5*) expression and its target genes *CCND2* (*cyclin D2*), *S6K1*, *E2F1*, *p27* ^*Cdkn1b*^, *p21* ^*Cdkn1a*^, *p57* ^*Cdkn1c*^, *PAK3*, and *FOS*. (a) mRNA relative gene expression in transfected cells with control siRNA (siC) and *ETV5* siRNA (siE). Student's *t*‐test, **P < 0.001, ***P < 0.0001, *n* = 4. (b) Immunoblot for ETV5, E2F1, and β‐Actin, in presence (siC) or *ETV5* partial absence (siE). (c) Protein levels analysis of ETV5 and E2F1 normalizing to β‐Actin. Student's *t*‐test, *P < 0.05, *n* = 3. (d) p27^Cdkn1b^ immunodetection in control siRNA (siC) and *ETV5* siRNA (siE) transfected cells. (e) Protein levels analysis of ETV5 and p27^Cdkn1b^ normalized to β‐Actin. Student's *t*‐test, *P < 0.05, *n* = 3. All experiments were analyzed at 72 h post‐transfection with siRNA control or siRNA *ETV5* (siC or siE). All results are presented as the mean ± SD.

In cells transfected with siE, *ETV5* expression decreased by ~ 80% (Fig. 3a). The expression of *CCND2* and *S6K1* was not modified in the presence or absence of *ETV5* (Fig. 3a). However, when *ETV5* is partially silenced after 72 h of transfection, it provoked an upregulation of the mRNA levels of *E2F1* (100%), *p27* ^*Cdkn1b*^ (43%), *p21* ^*Cdkn1a*^ (71%), and *p57* ^*Cdkn1c*^ (44%) inhibitors of the cyclin/CDKs complexes (Fig. 3a). On the contrary, *ETV5* silencing induced downregulation of cell cycle activators, *PAK3* (47%) and *FOS* (−54%) (Fig. 3a).

Little is known about the implications of *PAK3* and *FOS* in cell cycle. It has been shown that *FOS* controls *cyclin D1* transcription, hence, cell cycle [17]. Our results did not show any regulation on *cyclin D2* and no other cyclin D was differentially expressed in the ETV5 KO model or the human islets silencing. We, therefore, decided not to pursue this via.

On the contrary, we noticed that the three cyclin/CDK inhibitors analyzed were upregulated. It has previously been reported that ETV5 binds directly to *p21* ^*Cdkn1a*^ promoter, repressing its expression [19]. Moreover, *Etv4* and *Etv5* double knockout in embryonic stem cells produced decreased proliferation and enhanced p57 expression [20]. However, little is known about a possible implication of *ETV5* regulation on *p27* ^*Cdkn1b*^, and it has been reported that E2F1 induces overexpression of *p27* ^*Cdkn1b*^, provoking the arrest of the cell cycle [14, 24]. Therefore, we measured protein levels of E2F1 and p27^Cdkn1b^. We observed that upon *ETV5* silencing, ETV5 protein levels decreased by 21% compared with siC cells (Fig. 3b,c). We found that protein levels of *E2F1* increased 31% (Fig. 3b,c) and 28% for p27^Cdkn1b^ after the transfection with siE (Fig. 3d,e). These results suggest that partial absence of *ETV5* reduces cell proliferation via a mechanism that involves upregulation of E2F1 and *p27* ^*Cdkn1b*^.

## Discussion

The present work analyzed the involvement of ETV5 and its target genes on cell proliferation in pancreatic β cells, INS‐1 (832/13).

We have previously reported experiments of glucose‐stimulated insulin secretion with INS‐1 (832/13) in partial absence of ETV5, observing a decrease in insulin secretion, when cells were starved and then stimulated with high glucose concentrations for 1 h [3]. We then used the same silencing and transfection reagents in the same cellular model [INS‐1 (832/13)]. We observed that ETV5 mRNA silencing is reduced up to 80% after 72 h post‐transfection (Figs 1a or 3a), and around 21% for protein levels. This partial silencing is consistent with our previous report [3]. Despite this ETV5 partial silencing, we do observe a reduction in cell number, as well as in proliferation, and different expression level of its cell cycle target genes.

On the contrary, insulin is able to promote cell proliferation for its known action as a mitogen [21]. For this reason, secreted insulin concentration in the media was measured every 24 h up to 72 h after transfection, showing no differences between the siE or siC cells. At the same time, cell number and confluence of INS‐1 (832/13) cell culture were decreased at 72 h, while *ETV5* was partially silenced. Hence, the observed decrease in cell number after *ETV5* partially silencing cannot be a consequence of reduced insulin mitogen activity.

The effect of ETV5 on cell number and cell proliferation has previously been described elsewhere. In colorectal cancer model, the silencing of *ETV5* suppresses cell proliferation [25]. Additionally, in papillary thyroid cancer model, silencing of *ETV5* causes a decrease in cell proliferation and this was associated with a diminished expression of *CCND1* (*cyclin D1*) and *CCND2* (*cyclin D2*) [18]. Also, in a model of immortalized urothelial cells, presence or absence of ETV5 was correlated with the number of cells [26].

In the present work, evaluation of viability, apoptosis, and cell proliferation was performed. We found that *ETV5* downregulation reduced the cell number by decreasing cell proliferation, but no modification was observed on viability or apoptosis processes. Indeed, the ~ 32% decrease in cell number was consistent with the ~ 28.23% reduction in cell proliferation during the partial silencing of *ETV5*. These results reveal the implication of *ETV5* in proliferation of INS‐1 (832/13) β cells, which is essential for the maintenance of an adequate β‐cell mass *in vivo* [7], and would be interesting to observe in an *in vivo* model.

As we demonstrated that *ETV5* is involved in the cell proliferation of INS‐1 (832/13) cells, we then looked for genes involved in cell cycle that were reported to be differentially expressed in transcriptomic database of *ETV5 KO* mice or silenced *ETV5* human islets [4, 23].

Repeatedly, E2F1 has been reported to be involved in several types of cancer and regulate the transcription of genes of the S phase in the cell cycle [27]. For example, *ETV5* is overexpressed in synovial sarcoma tumors. Upon *ETV5* downregulation in the HS‐SY‐II and SYO‐1 cells, *E2F1*, *p21*, and *FOS* were differentially expressed [28]. However, E2F1 has also been described to have both effects: pro‐tumorogenic or antitumor in colon cancer [29], and these effects on proliferation depended on the type of cancer or cell line.

In our model, INS‐1 (832/13) cells, we demonstrated that partially silencing *ETV5* provoked *E2F1* overexpression. Therefore, when *ETV5* was downregulated, the repression over *E2F1* was released and upregulation of *E2F1* transcript was observed.

On the contrary, it is known that E2F1 can induce the expression of *p27* ^*Cdkn1b*^ by direct binding to its promoter region [14]. In a model of hepatic cells, it has been demonstrated that an overexpression of *E2F1* triggers increased expression of *p27* ^*Cdkn1b*^, which is well known to be an inhibitor of the cell cycle [30]. Also, E2F1 can directly bind to *p21* ^*Cdkn1a*^ promoter and induce protein levels, regulating cell cycle arrest [15]. Our results demonstrate that upon *ETV5* silencing, *E2F1*, *p27* ^*Cdkn1b*^, and p21^Cdkn1a^ were upregulated in the INS‐1 (832/13) cells, provoking cell arrest. This suggests a transcriptional regulation of *ETV5* on *E2F1*, but in an opposite direction than the one established in the synovial sarcoma report [28].

Recently, it was reported that *ETV5* silencing in human islets resulted in an upregulation of *p57* ^*Cdkn1c*^ [4]. Consistently, we demonstrated that *ETV5* partially silenced in the INS‐1 (832/13) cells, induced overexpression of *p57* ^*Cdkn1c*^. Moreover, *Etv4* and *Etv5* double knockout in embryonic stem cells produced decreased proliferation and enhanced *p57* expression [20]. Together, these results indicate that *ETV5* silencing reduces cell proliferation via the upregulation of genes involved in cell cycle arrest (*p21* ^*Cdkn1a*^, *p27* ^*Cdkn1b*^, and *p57* ^*Cdkn1c*^), inhibitors of the cyclin/CDK complexes.

PAK3 has been reported to regulate β‐cell proliferation and differentiation in β cells in embryonic pancreas, and it is necessary to maintain glucose homeostasis in adult mice [16]. Our results demonstrate that *PAK3* expression is downregulated in *ETV5* silenced cells, which is consistent with previous findings in human islets, where *ETV5* knockdown resulted in decreased *PAK3* expression [4].

Another gene is the protooncogene *FOS* that controls cell cycle reentry or progression [17]. Here, we show that the downregulation of *ETV5* promotes decreased expression of *FOS* and the same outcome was found in human islets, where *ETV5* knockdown was associated with lower *FOS* mRNA levels [4]. The overexpression of *FOS* induces the activation and expression of several factors that promote β‐cell proliferation, insulin secretion, and cellular survival [31]. *FOS* regulates the transcription of *cyclin D1* [17], and it is well established that this cyclin promotes the progression of the G1 to the S phase. Our results showed no effect on *cyclin D2* expression, but it is possible that ETV5 controls cell proliferation through the action of *FOS* and its influence on *cyclin D1* expression.

**Fig. 4:**
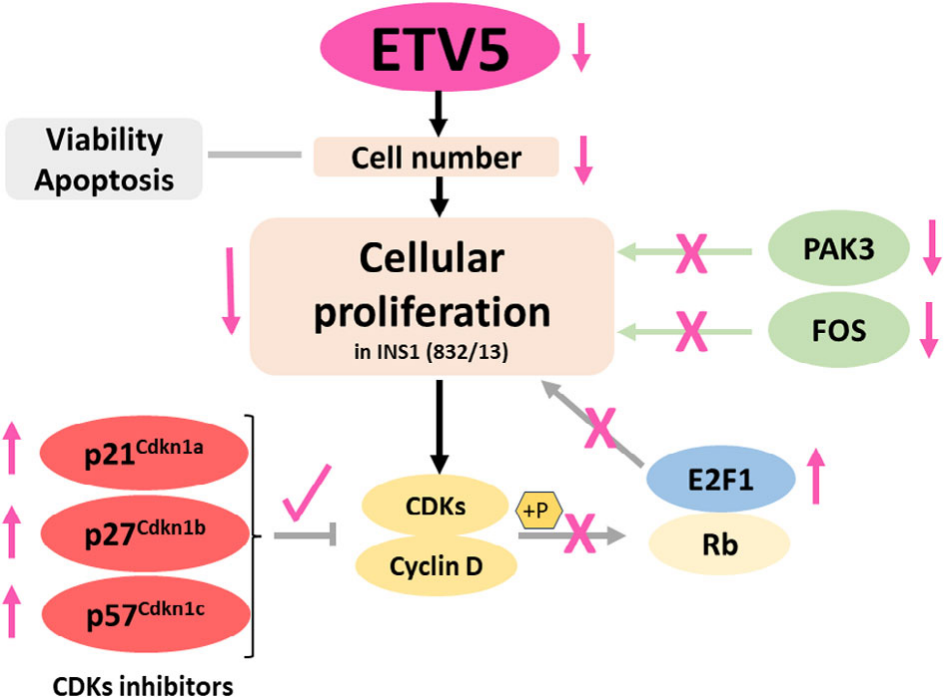
E‐twenty‐six variant 5 (ETV5) partial silencing modulates cell proliferation in INS‐1 (832/13) without modifying apoptosis or viability. ETV5 regulates cell proliferation by increasing gene expression of inhibitors of the cyclin/CDKs complexes (*p21* ^*Cdkn1a*^, *p27* ^*Cdkn1b*^, and *p57* ^*Cdkn1c*^) and *E2F1*, and downregulation of cell cycle activators (*PAK3* and *FOS*).

In summary, this work is novel because it demonstrates that the partial absence of *ETV5* in INS‐1 (832/13) cell line decreases cell proliferation independently of insulin secretion, via a mechanism that involves upregulation of *E2F1* and inhibitors of the cyclin/CDKs complexes (*p21* ^*Cdkn1a*^, *p27* ^*Cdkn1b*^, and *P57* ^*Cdkn1c*^) and downregulation of cell cycle activators (*PAK3* and *FOS*), summarized in Fig. 4. The latter suggests a new function of ETV5 in maintaining a correct cell number and possibly contributing to pancreas functionality and metabolic health.

## Conflict of interest

The authors declare no conflict of interest.

### Peer review

The peer review history for this article is available at https://www.webofscience.com/api/gateway/wos/peer‐review/10.1002/2211‐5463.13724.

## Author contributions

YED‐L contributed to the acquisition and analysis of the data and wrote the manuscript. GEP‐F contributed to acquisition of data with MUSE cell analyzer and revised the article. VC‐D and MEF contributed to the acquisition of data. MEF revised the article. RG‐A contributed to the conception and design of the study, funding, analysis, and interpretation of the data and drafted/revised the article. RG‐A is the corresponding author and guarantor.

## Data Availability

The data that support the findings of this study are available from the corresponding author upon request.
